# A novel prognostic model based on four circulating miRNA in diffuse large B‐cell lymphoma: implications for the roles of MDSC and Th17 cells in lymphoma progression

**DOI:** 10.1002/1878-0261.12834

**Published:** 2020-11-09

**Authors:** Rui Sun, Zhong Zheng, Li Wang, Shu Cheng, Qing Shi, Bin Qu, Di Fu, Christophe Leboeuf, Yan Zhao, Jing Ye, Anne Janin, Wei‐Li Zhao

**Affiliations:** ^1^ Shanghai Institute of Hematology State Key Laboratory of Medical Genomics National Research Center for Translational Medicine at Shanghai Ruijin Hospital Affiliated to Shanghai Jiao Tong University School of Medicine China; ^2^ Laboratory of Molecular Pathology Pôle de Recherches Sino‐Français en Science du Vivant et Génomique Shanghai China; ^3^ Department of Laboratory Medicine Shanghai Rui Jin Hospital Shanghai Jiao Tong University School of Medicine China; ^4^ U1165 Inserm/Université Paris 7 Hôpital Saint Louis Paris France

**Keywords:** diffuse large B‐cell lymphoma, microRNA, prognosis, Ras protein signal transduction, tumor microenvironment

## Abstract

MicroRNA (miRNA) have been emerged as prognostic biomarkers in diffuse large B‐cell lymphoma (DLBCL). To understand the potential underlying mechanisms and translate these findings into clinical prediction on lymphoma progression, large patient cohorts should be evaluated. Here, using miRNA PCR array, we analyzed the miRNA expression profiles in serum samples of 20 DLBCL patients at diagnosis, remission and relapse. Four candidate miRNA were identified and subsequently evaluated for their ability to predict relapse and survival. A prognostic model based on four circulating miRNA (miR21, miR130b, miR155 and miR28) was established and tested in a training cohort of 279 patients and in a validation cohort of 225 patients (NCT01852435). The prognostic value of the 4‐circulating miRNA model was assessed by univariate and multivariate analyses. The novel 4‐circulating miRNA prognostic model significantly predicted clinical outcome of DLBCL, independent of International Prognostic Index in the training cohort [hazard ratio (HR) = 2.83, 95% CI 2.14–3.51, *P* < 0.001] and in the validation cohort (HR = 2.71, 95% CI 1.91–3.50, *P* < 0.001). Moreover, DNA‐ and RNA‐sequencing was performed on tumor samples to detect genetic mutations and signaling pathway dysregulation. DNA‐sequencing data showed no significant difference of tumor mutation burden between the low‐risk and the high‐risk groups of the 4‐circulating miRNA model. RNA‐sequencing revealed a correlation between the 4‐circulating miRNA model and aberrant Ras protein signaling transduction. The impact of the miRNA signature on oncogenic signaling and tumor microenvironment was analyzed *in vitro* and *in vivo*. In B‐lymphoma cells, modulation of the miRNA regulated IGF1 and JUN expression, thereby altering MDSC and Th17 cells. In DLBCL patients, the high‐risk group presented Ras signaling activation, increased MDSC and Th17 cells, and immunosuppressive status compared with the low‐risk group. In conclusion, the easy‐to‐use 4‐circulating miRNA prognostic model effectively predicted relapse and survival in DLBCL. Moreover, the tumor microenvironment contributes to the role of the 4‐circulating miRNA model in DLBCL progression.

## Graphical abstract

A novel prognostic model based on four circulating miRNA (miR21, miR155, miR130 and miR28) showed independent prognostic significance in diffuse large B‐cell lymphoma. The 4‐circulating miRNA signature correlated with aberrant Ras protein signal transduction via IGF1 and JUN expression, and MDSC‐ and Th17 cell‐dependent lymphoma progression.

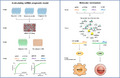

**Abbreviations**
AUC: areas under the curve
cDNA: complementary DNA
DLBCL: diffuse large B‐cell lymphoma
IGF1: insulin‐like growth factor 1
IL: interleukin
IPI: International Prognostic Index
JNK: JUN N‐terminal kinases
MDSC: myeloid‐derived suppressor cells
M‐HOPES: Multi‐center Hematology‐Oncology Programs Evaluation System
miRNA: microRNA
mRNA: messenger RNA
OS: overall survival
PBMC: peripheral blood mononuclear cells
PFS: progression‐free survival
TF: transcriptional factors
TIP: tumor immunophenotype
WES: whole‐exome sequencing
WGS: whole‐genome sequencing
WHO: World Health Organization

## 1 Introduction

Diffuse large B‐cell lymphoma (DLBCL) represents the most common neoplastic disorder of B‐lymphocytes [1]. Although conventional immunochemotherapy R‐CHOP (rituximab, cyclophosphamide, doxorubicin, vincristine and prednisolone) significantly improves the clinical outcome of DLBCL, patients with relapsed or refractory disease have a poor prognosis [2]. Molecular heterogeneity contributes to diverse outcomes of DLBCL, and accurate prediction of relapse and survival at diagnosis is critical for conducting risk‐adapted therapeutic strategies [3, 4]. Identifying high‐risk patients with easy‐to‐use circulating prognostic biomarkers thus remain of great importance in DLBCL.

MicroRNA (miRNA) belong to a class of 19‐ to 23‐nucleotide non‐coding RNA molecules and regulate gene expression by targeting messenger RNA (mRNA) at the 3′‐untranslated region. Multiple miRNA play an important role in lymphocyte development and malignant transformation, including miR21, miR130b, miR148a, miR155 and miR181 [5, 6]. In B‐ and T‐cell lymphomas, our studies and the others showed that aberrant expression of miR21, miR‐17/92 clusters, miR28, miR155 and miR181a was significantly associated with chemotherapy resistance or inferior survival [7, 8, 9, 10, 11]. However, no circulating miRNA prognostic model has been established and validated in large cohorts of DLBCL.

Ras protein signal transduction is essential for tumor cell growth, differentiation and survival. Gain‐of‐function mutations in Ras isoforms (H‐Ras, K‐Ras, N‐Ras) that directly activate Ras signaling have been identified in lymphomas [12]. In addition, Ras cascade provokes cancer progression by acting on immunosuppressive components within the tumor microenvironment, including myeloid‐derived suppressor cells (MDSC), Th17 cells, regulatory T cells and macrophages [13, 14]. IGF1 (insulin‐like growth factor 1) and JUN N‐terminal kinases (JNK) are major conserved pathways involved in Ras protein signal transduction and are overexpressed in DLBCL [15, 16].

In the present study, we assessed the expression patterns of miRNA during disease progression and developed a novel 4‐circulating miRNA prognostic model in DLBCL. Meanwhile, we explored the underlying biological mechanism and showed a potential impact of the 4‐circulating miRNA model on oncogenic pathway and tumor microenvironment.

## 2 Patients and methods

### 2.1 Patients

Patients with newly diagnosed DLBCL were enrolled in this study, including a discovery cohort of 20 patients with a series of serum samples at diagnosis, remission and at relapse, a training cohort of 279 patients received R‐CHOP retrospectively in a single Institute between 24 August 2012 and 21 April 2018, and a validation cohort of 225 patients received R‐CHOP50, R‐CEOP70 or R‐CEOP90 prospectively from a multi‐center, randomized clinical trial (NCT01852435) of the Multi‐center Hematology‐Oncology Programs Evaluation System (M‐HOPES) in China between 15 May 2013 and 29 February 2016 [17]. Clinical characteristics of the training and validation discovery cohorts of DLBCL patients are listed in Tables 1 and S1. Histological diagnosis was established according to World Health Organization (WHO) classification [18]. The study was approved by the Institutional Review Boards of all M‐HOPES centers, with informed consent obtained from all patients in accordance with the Declaration of Helsinki.

**Table 1:** Clinical characteristics and univariate analysis for predictors of PFS and OS in the training and validation cohorts of patients with DLBCL.

Characteristics	Training cohort (*n* = 279)	Validation cohort (*n* = 225)	*P*‐value	Training cohort		Validation cohort	
				*P*‐value for PFS	*P*‐value for OS	*P*‐value for PFS	*P*‐value for OS
Sex							
Female	138/279 (49.5%)	98/225 (43.6%)	0.186	0.098	0.382	0.765	0.637
Male	141/279 (50.5%)	127/225 (56.4%)					
Age							
> 60 years	129/279 (46.2%)	86/225 (38.2%)	0.071	0.052	0.003	0.125	0.006
≤ 60 years	150/279 (53.8%)	139/225 (61.8%)					
ECOG							
0–1	206/279 (73.8%)	202/225 (89.8%)	< 0.001	0.016	0.028	< 0.001	< 0.001
2	73/279 (26.2%)	23/225 (10.2%)					
Ann Arbor							
I–II	135/279 (48.4%)	134/225 (59.6%)	0.012	< 0.001	< 0.001	0.001	0.001
III–IV	144/279 (51.6%)	91/225 (40.4%)					
Extranodal involvement							
No	105/279 (37.6%)	163/225 (72.4%)	< 0.001	0.937	0.584	0.343	0.357
Yes	174/279 (62.4%)	62/225 (27.6%)					
LDH							
Normal	125/279 (44.8%)	145/225 (64.4%)	< 0.001	< 0.001	0.001	< 0.001	< 0.001
Elevated	154/279 (55.2%)	80/225 (35.6%)					
International Prognostic Index (IPI)							
0–2	151/279 (54.1%)	168/225 (74.7%)	< 0.001	< 0.001	< 0.001	< 0.001	< 0.001
3–5	128/279 (45.9%)	57/225 (25.3%)					
4‐circulating miRNA prognostic model							
Low risk	152/279 (54.5%)	165/225 (73.3%)	< 0.001	< 0.001	< 0.001	< 0.001	< 0.001
High risk	127/279 (45.5%)	60/225 (26.7%)					

### 2.2 MicroRNA array and real‐time PCR

Serum samples of the discovery cohort were analyzed for miRNA array, and those of the training and validation cohorts for quantitative PCR assessment of miRNA predictors. Total serum miRNA was extracted by miRNeasy Serum/Plasma Kit (Qiagen, Valencia, CA, USA). Serum miRNA expression was measured by real‐time polymerase chain reaction (PCR) using miScript reverse transcription Kit (Qiagen), miscript SYBR Green PCR Kit (Qiagen) and miScript miRNA PCR Arrays (MIHS‐3106ZE; Qiagen). MiR39 (MS00019789; Qiagen) was used as endogenous control. The reactions were analyzed on 7500HT Fast Real‐time PCR system (Applied Biosystems, Carlsbad, CA, USA). Real‐time PCR was performed under the following conditions: 95 °C 15 min; 94 °C 15 s, 55 °C 30 s and 70 °C 30 s (40 cycles). A relative quantification was calculated using the 2^−ΔΔCT^ method.

### 2.3 DNA‐sequencing

Tumor samples of 223 patients were analyzed for gene mutations using whole‐genome/exome sequencing (WGS/WES, *n* = 177) or targeted sequencing (*n* = 46). Whole‐exome sequencing (WES) was performed on frozen tumor tissue and FFPE tumor tissue quality‐controlled by agarose gel electrophoresis. Whole‐genome sequencing (WGS) was performed on frozen tumor tissue. Targeted sequencing was performed on FFPE tumor tissue using a panel of 135 recurrently mutated genes based on WES and WGS results. Mutation frequencies per gene and mutation signatures revealed no significant difference in the results for WES, WGS and targeted sequencing.

### 2.4 RNA‐sequencing

Tumor samples of 52 patients were analyzed for gene expression profile and tumor immunophenotype (TIP) using RNA‐sequencing. Total RNA was extracted using TRIzol and RNeasy MinElute Cleanup kit from frozen tissue samples. Following extraction, RNA quantity was evaluated on Nanodrop and the integrity of total RNA using RNA 6000 Nano Kit on an Agilent 2100 Bioanalyzer (Shanghai Asiagene Technology Co., Ltd, Shanghai, China). The RNA library was constructed using TruSeq RNA Sample Preparation Kit. The poly‐A containing‐mRNA molecules were purified using oligo‐dT attached magnetic beads. Following purification, the mRNA was fragmented into small pieces using divalent cations under elevated temperature. The cleaved RNA fragments were copied into first strand complementary DNA (cDNA) using reverse transcriptase and random primers, followed by second strand cDNA synthesis using DNA Polymerase I and RNase H. The cDNA fragments went through an end repair process, the addition of a single ‘A’ base, and ligation of the adapters. The products were purified and enriched with PCR to create the final cDNA library. The clusters of the cDNA library were generated on the flow cell using TruSeq PE Cluster Kit (Illumina, San Diego, CA, USA) and HiSeq PE flow cell and sequenced on the HiSeq 2000 system using the TruSeq SBS Kit (Illumina).

The mean read count of each sample was 91 630 631, with an average of 90.9% Q30 Bases (range 87.3–93.0%). Reading pairs were aligned to Refseq hg19 (downloaded from UCSC Genome Browser, http://hgdownload.soe.ucsc.edu/) by star (v2.5.2b) according to the Genome Analysis Toolkit (gatk, v3.7.0, Broad Institute, Cambridge, MA, USA) recommended pipeline. Transcript count table files were generated by the HTSeq using the GENCODE annotation database and processed with the BAM files generated by hisat2 (Bloomberg School of Public Health, Baltimore, MD, USA). limma version 3.34.9 (Australia Department of Mathematics and Statistics, Parkville, Australia) was used to normalize the raw reads and obtain differentially expressed genes. DEG were then analyzed by the Database for Annotation, Visualization and Integrated Discovery (DAVID) v6.8 (https://david.ncifcrf.gov/) and were enriched in Gene ontology pathways. Gene Set Enrichment Analysis (GSEA) was performed using the gsea (v2.2.3, http://software.broadinstitute.org/gsea/downloads.jsp) with MSigDB‐curated gene sets (c5.bp.v6.2.symbols.gmt).

### 2.5 Tumor immunophenotype analysis

RNA‐sequencing data was used to perform tracking tumor immunophenotype (TIP, http://biocc.hrbmu.edu.cn/TIP/index.jsp) analysis, focused on profiling immune microenvironment based on the seven‐step cancer‐immunity cycle and inferring the proportion of various tumor‐infiltrating immune cells.

### 2.6 Ratio model training

MicroRNA array data from serum samples of 20 DLBCL patients were collected. Of 372 serum miRNA analyzed, we first focused on the eight miRNA that were significantly altered between diagnosis and remission and between relapse and remission. Secondly, to ensure robustness, we selected four of the most significant miRNA (miR21, miR130b, miR155 and miR28) for model establishment, after validation in serum samples of 20 relapse patients and 80 non‐relapse patients of the training cohort.

To investigate the effectiveness of the 4‐circulating miRNA prognostic model, we assigned a risk score for each patient according to a linear combination of the expression level of the miRNA. The risk score function (RSF) for sample *i* using the information from the significant miRNA was calculated as follows:${\text{RSF}}_{i}=\sum_{j=1}^{k}W_{j}\times S_{\mathrm{ij}}$. In the above equation, *S_ij_* is the risk score for miRNA *j* on sample *i*, and *W_j_* is the weight of the risk score of miRNA *j*. Weights were obtained by the coefficients derived from the logistic regression analyses. Patients were divided into low‐risk and high‐risk groups using the median miRNA signature risk score as the cutoff point. To evaluate the prognostic potential of individual miRNA and 4‐circulating miRNA models, receiver operating characteristic (ROC) curves were generated with areas under the curve (AUC) calculated.

### 2.7 *In vitro* co‐culture system

B‐lymphoma cell line OCI‐LY10 was obtained from American Type Culture Collection (Manassas, VA, USA). Peripheral blood mononuclear cells (PBMC) were isolated from peripheral blood, as previously described [11]. Cells were co‐cultured in a humidified atmosphere of 95% air and 5% CO_2_ at 37 °C using Transwell culture chambers (8 μm; Millipore Corporation, Billerica, MA, USA). In the co‐culture system, lymphoma cells were plated on the upper chamber, with immune cells on the lower chamber, allowing direct contact of lymphoma cells with immune cells. OCI‐LY10 cells were transfected with miR21 inhibitor, miR130b inhibitor, miR155 inhibitor, miR28 mimics (Riobio, Guangzhou, China) or negative control (Riobio) using lipofectamine 2000 (Invitrogen, Carlsbad, CA, USA) following the manufacturer's instructions.

### 2.8 Flow cytometry

To detect MDSC percentage, co‐cultured cells were stained with anti‐CD45, CD3, CD56, HLA‐DR, CD116, CD33 and CD19 (Becton Dickinson, Franklin Lakes, NJ, USA) for 30 min. To detect the percentage of Th17 cells, co‐culture cells were stimulated with 10 ng·mL^−1^ PMA and 1 µg·mL^−1^ ionomycin (Millipore) in the presence of 1 µg·mL^−1^ brefeldin A (Biolegend, San Diego, CA, USA) for 6 h. Cells were washed in FACS buffer (PBS, 2% FCS and 0.02% NaN_3_). Cells were stained with anti‐CD45, CD4 (Becton Dickinson) for 30 min, treated with Fixation/Permeabilization solution (Becton Dickinson) for 40 min, and acquired using FACS Canto and LSR‐II flow cytometers (Becton Dickinson) after staining with anti‐interleukin (IL)‐17 (Becton Dickinson) for 15 min. Data were analyzed using flowjo software (Becton Dickinson).

### 2.9 Real‐time PCR

Total RNA was extracted from OCI‐LY10 cells using TRIzol reagent (Invitrogen). cDNA was synthesized using PrimeScript RT reagent Kits with gDNA Eraser (TaKaRa, Visalia, CA, USA) following the manufacturer's instructions. Real‐time PCR was performed using SYBR Premix Ex TaqTM II (TaKaRa) on ABI Prism 7500 (Applied Biosystems, Bedford, MA, USA). The relative gene expression levels were calculated using the sds2.4 software (Invitrogen). The primer sequences were as follows: IGF1 forward primer: 5′‐GCTCTTCAGTTCGTGTGTGGA‐3′; reverse primer: 5′‐GCCTCCTTAGATCACAGCTCC‐3′. JUN forward primer: 5′‐TCCAAGTGCCGAAAAAGGAAG‐3′; reverse primer: 5′‐CGAGTTCTGAGCTTTCAAGGT‐3′. GAPDH forward primer: 5′‐GAAGGTGAAGGTCGGAGTC‐3′; reverse primer: 5′‐GAAGATGGTGATGGGATTTC‐3′. GAPDH was used as the endogenous control and DB cells for calibration. A relative quantification was calculated using the 2^−ΔΔCT^ method.

### 2.10 Luciferase report assay

Total cDNA from HEK‐293T cells was used to amplify the promoter region (−674 to −667 bp) of IGF1, forward primer: 5′‐ATCTG TTCCGCGTGGATGAAGGGAGACAGCAGACATCTGAATG‐3′; reverse primer: 5′‐TCACGATGCGGCCGCTCGAGTATTCACAGGCAAAGTAGTCCTTCAAG‐3′. The *Bam*HI and *Xho*I restriction enzyme sites were used. HEK‐293T cells were seeded in 24‐well plates and co‐transfected with 100 nm of miR130b mimics, 100 ng·mL^−1^ promoter region (−674 to −667 bp) luciferase reporter construct, and 10 ng·mL^−1^ luciferase reporter using lipofectamine 2000. Cells were collected 24 h after transfection, using the Passive Lysis Buffer (30 μL per well) provided as part of the Dual‐Luciferase Reporter Assay System Kit (Promega, Madison, WI, USA). Firefly and Renilla luciferase activities were examined by the Dual‐Luciferase Reporter Assay System and detected by a Centro XS3 LB960 Luminometer (Berthold, Oak Ridge, TN, USA).

### 2.11 Western blot

Cells were collected and lysed in 200 μL lysis buffer (Sigma Aldrich, Shanghai, China). Protein lysates (20 μg) were electrophoresed on 10% sodium dodecyl sulfate polyacrylamide gels and transferred to nitrocellulose membranes. Membranes were blocked with 5% non‐fat dried milk and incubated overnight at 4 °C with appropriate primary antibody, followed by horseradish peroxidase‐linked secondary antibody. The immunocomplexes were visualized using chemiluminescence phototope‐horseradish peroxidase kit. Anti‐IGF1 antibody was from Abcam (Cambridge, UK) (ab40657) and anti‐JUN antibody from Cell Signaling Technology (CST14537825, Danvers, MA, USA). Anti‐β‐actin antibody was from Proteintech (HRP‐6008, Manchester, UK) to ensure equivalent loading of cell protein.

### 2.12 Immunohistochemistry and immunofluorescence assay

Immunohistochemistry was performed on 5‐μm paraffin sections with an indirect immunoperoxidase method using the primary antibody against IGF1 (1 : 400; Abcam) and JUN (1 : 400; Cell Signaling Technology). Scores for percentage of IGF1‐positive cells or JUN‐positive cells and scores for expression intensities were multiplied to calculate an immunoreactive score (IRS): + ~ ++ = no staining or weak staining; +++ ~ ++++ = moderate staining to strong staining. Immunofluorescence assay was performed on methanol‐fixed cells using antibody against IGF1 (1 : 100; Abcam) and JUN (1 : 100; Cell Signaling Technology). Texas Red conjugated donkey anti‐rabbit IgG antibody (Cell Signaling Technology) and FITC‐conjugated goat anti‐mouse IgG (Cell signaling) were used as the secondary antibody.

### 2.13 Statistical analysis

Differences of miRNA expression among groups were assessed by Mann–Whitney *U*‐test. *In vitro* experimental results were indicated as mean ± SD of data obtained from three separate experiments and determined by *t*‐test to compare variance. Progression‐free survival (PFS) was calculated from the date when treatment began, to the date when the disease progression was recognized or the date of last follow‐up. Overall survival (OS) was calculated from the date of diagnosis to the last follow‐up or the date of death. Univariate hazard estimates (HR) were generated with unadjusted Cox proportional hazards models. Covariates demonstrating statistical significance with *P*‐values < 0.05 on univariate analyses were included in the multivariate model. Statistical procedures were performed with spss version 20.0 (SPSS Inc, Chicago, IL, USA) statistical software package or graphpad prism 5 software (GraphPad company, San Diego, CA, USA). *P* < 0.05 was considered statistically significant.

## 3 Results

### 3.1 Establishment of a 4‐circulating miRNA prognostic model in DLBCL

**Fig. 1:**
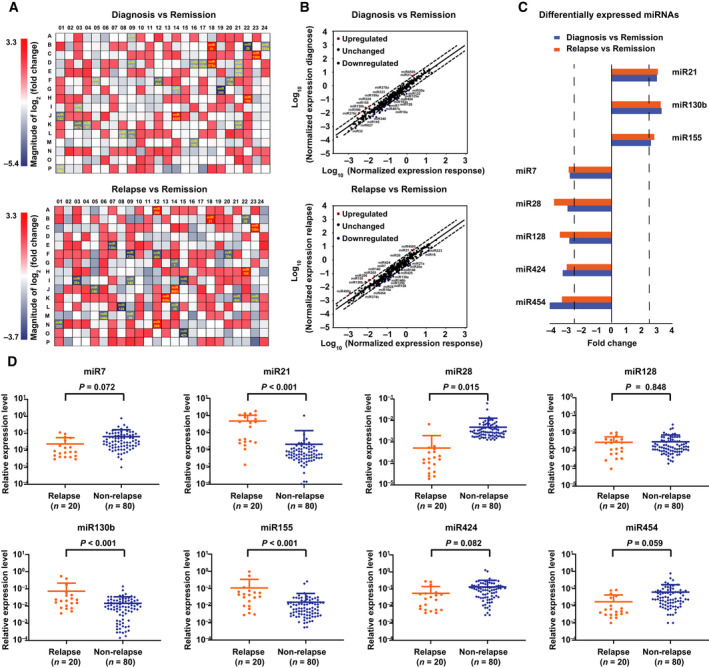
Establishment of a 4‐circulating miRNA prognostic model in DLBCL. (A) Serum miRNA expression fold change calculated between diagnosis vs remission, and relapse vs remission in DLBCL (*n* = 20) by real‐time PCR. The relative expression level of each sample was calculated based on the lowest expression value. (B) Differential expression of each miRNA at diagnosis vs remission, and relapse vs remission. (C) The fold change of miRNAs significantly altered at diagnosis or at relapse. (D) Validation of the above miRNAs in relapse patients (*n* = 20) and non‐relapse DLBCL (*n* = 80).

To evaluate systematically the circulating miRNA signatures and determine their relationship with lymphoma relapse, miRNA expression profiling was revealed by miRNA PCR array of 372 miRNA in serum samples of 20 DLBCL patients at diagnosis, remission and relapse (Fig. 1A). Compared with serum miRNA levels in remission, five miRNA were significantly increased and 23 miRNA decreased at diagnosis, and seven miRNA increased and 18 miRNA decreased at relapse (Fig. 1B). The inclusion criteria of miRNA for further validation was lymphoma‐associated miRNA upregulated (miR21, miR130b and miR155) or downregulated (miR7, miR28, miR128, miR424 and miR454) with a mean fold change > 2.5 and a *P*‐value < 0.05 between diagnosis and remission, or between relapse and remission (Fig. 1C). We then investigated the expression of these miRNA using real‐time quantitative PCR in serum samples of another 20 relapsed and 80 non‐relapsed patients of the training cohort (Fig. 1D). Serum miR21, miR130b and miR155 were significantly upregulated, whereas miR28 was downregulated in relapsed patients (*P* < 0.001, *P* < 0.001, *P* < 0.001 and *P* = 0.015, respectively), all of which were utilized to generate a 4‐circulating miRNA prognostic model in DLBCL.

### 3.2 Association of the 4‐circulating miRNA prognostic model with disease relapse

**Fig. 2:**
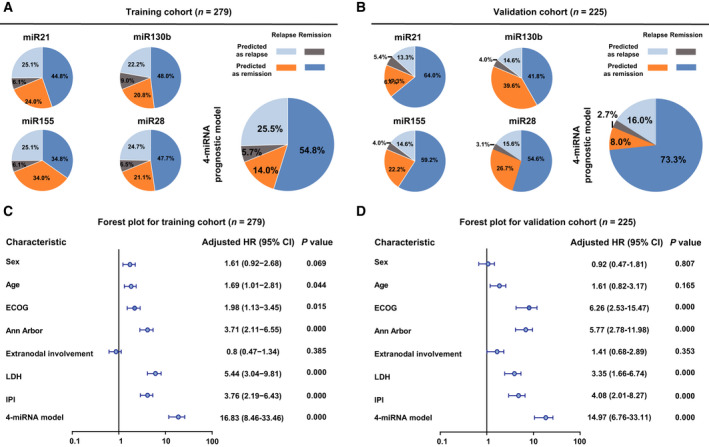
Association of the 4‐circulating miRNA prognostic model with disease relapse. (A,B) The true‐positive rate, true non‐positive rate, false‐positive rate and false non‐positive rate of each miRNA and 4‐circulating miRNA model in the training cohort (A) and validation cohort (B). (C,D) Forest plot showing the distribution of clinical features in terms of relapse in the training cohort (C) and validation cohort (D).

To determine the association of the 4‐circulating miRNA model with disease relapse, we performed ROC analysis in the training and validation cohorts of DLBCL. Each miRNA and 4‐circulating miRNA prognostic model was tested for prediction accuracy of relapse and remission. The true‐positive rate, true non‐positive rate, false‐positive rate and false non‐positive rate of each miRNA and 4‐circulating miRNA prognostic model are displayed in the training cohort (Fig. 2A) and validation cohort (Fig. 2B). Although clinical features were different between these two cohorts, the true‐positive rate and true non‐positive rate were significantly higher in the 4‐circulating miRNA model than in each miRNA.

We used univariable analyses and forest plots to visualize the distribution of clinical features [sex, age, ECOG, Ann Arbor stage, extranodal involvement, serum LDH, international prognostic index (IPI) and 4‐circulating miRNA model] in terms of relapse status. The 4‐circulating miRNA model was significantly correlated with an increased risk of relapse both in the training cohort (HR = 16.83, *P* < 0.001, Fig. 2C) and in the validation cohort (HR = 14.97, *P* < 0.001, Fig. 2D).

### 3.3 Association of the 4‐circulating miRNA prognostic model with survival time

**Fig. 3:**
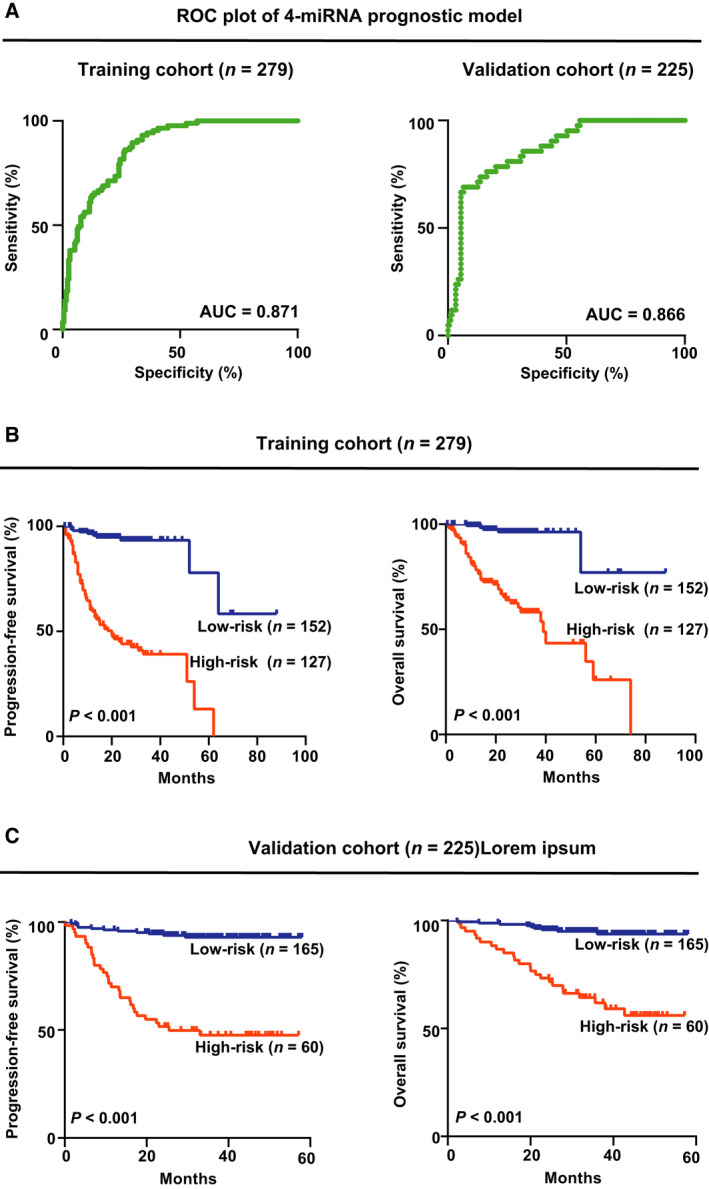
Association of the 4‐circulating miRNA prognostic model with survival time. (A) ROC curve of the training cohort and the validation cohort. (B) Survival curve of progression‐free survival (PFS) and overall survival (OS) in the training cohort according to the low‐risk and the high‐risk group of the 4‐circulating miRNA model. (C) Survival curve of PFS and OS in the validation cohort according to the low‐risk and the high‐risk of the 4‐circulating miRNA model.

As illustrated by ROC curve, areas under the curve (AUC) were 0.871 in the training cohort and 0.866 in the validation cohort (Fig. 3A). A cutoff prognostic model of −1.183 predicted the risk of relapse with the highest accuracy (89.1% sensitivity and 82.4% specificity). Risk score was used to differentiate low‐risk and high‐risk of relapse based on the weighted coefficients in the 4‐circulating miRNA model.

For prognostic evaluation, in the training cohort, the median follow‐up time was 21.0 months (range: 0.2–88.0 months), with a 2‐year PFS and OS of the patients of 64.4% and 79.4%, respectively. By univariate analyses, the 2‐year PFS and OS were 34.5% and 62.4% for high‐risk patients, significantly shorter than those of low‐risk patients of the 4‐circulating miRNA model (91.4% and 95.0%, both *P* < 0.001, Fig. 3B). In the validation cohort, the median follow‐up time was 35.0 months (range: 0.2–58.0 months), with a 2‐year PFS and OS of the patients of 83.0% and 89.7%, respectively. By univariate analyses, the 2‐year PFS and OS were 51.7% and 73.3% for high‐risk patients, significantly shorter than those of low‐risk patients of the 4‐circulating miRNA model (94.5% and 95.7%, both *P* < 0.001; Fig. 3C).

Using multivariate analyses, controlled by IPI, the 4‐circulating miRNA prognostic model was significantly associated with increased relapse rate, as well as inferior PFS and OS, both in the training cohort (all *P* < 0.001) and in the validation cohort (all *P* < 0.001, Table 2).

**Table 2:** Multivariate analysis of predictors of relapse and survival in DLBCL.

Variable	HR	Training cohort (*n* = 279) (95% CI)	*P*‐value	HR	Validation cohort (*n* = 225) (95% CI)	*P*‐value
Relapse						
IPI	1.323	0.786–1.861	< 0.001	1.407	0.701–2.113	< 0.001
4‐circulating miRNA prognostic model	2.823	2.135–3.510	< 0.001	2.706	1.912–3.500	< 0.001
PFS						
IPI	1.977	1.248–3.131	0.004	2.168	1.168–4.024	0.014
4‐circulating miRNA prognostic model	10.226	5.383–19.426	< 0.001	8.495	4.198–17.187	< 0.001
OS						
IPI	2.099	1.186–3.715	0.011	2.290	1.138–4.607	0.020
4‐circulating miRNA prognostic model	8.013	3.606–17.806	< 0.001	6.852	3.126–15.018	< 0.001

### 3.4 Association of the 4‐circulating miRNA prognostic model with genomic alterations

The mutational pattern of 63 genes recurrently and functionally mutated in DLBCL is shown in 223 patients, including 136 low‐risk patients and 87 high‐risk patients of the 4‐circulating miRNA model (Fig. S1, available online). A total of 806 somatic mutations of 63 genes were identified in 205 of 223 (91.9%) patients. A similar gene mutation pattern was presented in low‐risk and high‐risk patients, as classified into four categories: immune response, signaling pathway, chromatin organization and unclassified (Fig. S1A). According to each miRNA and the 4‐circulating miRNA model, no significant difference of tumor mutation burden was observed, as calculated by the number of the mutations involved in the four categories (Fig. S1B). In addition, since transcriptional factors (TF) regulated miRNA expression, we performed intersected network analysis to determine the relationship between TF and miRNA. Among the 63 genes, 20 TF were identified that may regulate the biogenesis of at least one miRNA (TransmiR database, Fig. S1C). However, according to each miRNA and the 4‐circulating miRNA model, no significant difference of TF gene mutation was observed (Fig. S1D), indicating that the 4‐circulating miRNA model was not determined by frequent mutation of TF in DLBCL.

### 3.5 Association of the 4‐circulating miRNA prognostic model with oncogenic signaling pathways

**Fig. 4:**
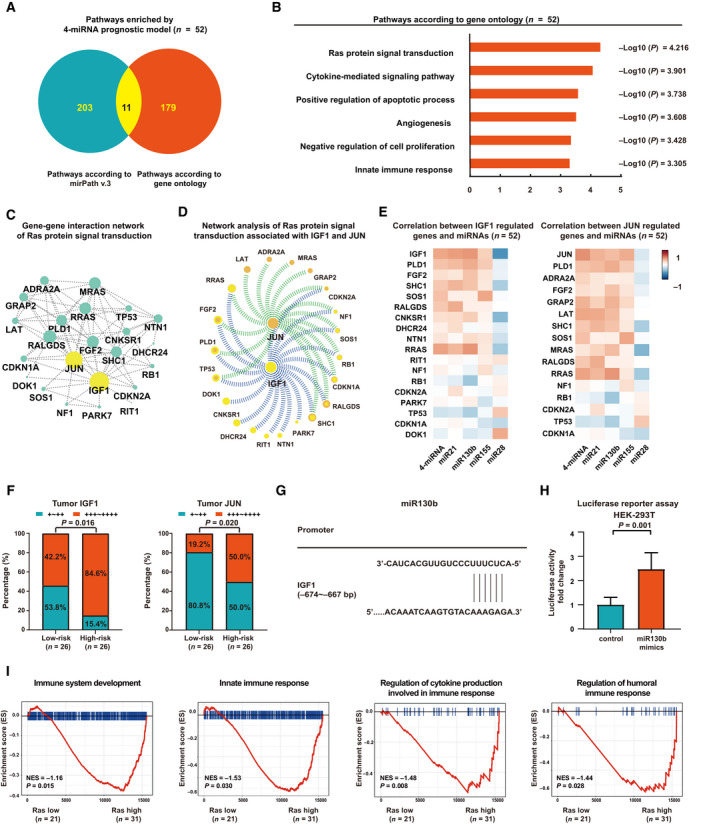
Association of the 4‐circulating miRNA prognostic model with oncogenic signaling pathways. (A) Signaling pathways regulated by four miRNA according tomirpathv.3 and signaling pathways enriched through gene ontology. (B) Lymphoma‐associated pathways identified in gene ontology. (C) Gene–gene interaction network of Ras protein signal transduction. Greater nodes indicated genes that were more likely to be functionally related. (D) Network analysis of Ras protein signal transduction associated with IGF1 and JUN. (E) Correlation coefficient between genes involved in Ras protein signal transduction and each miRNA, 4‐circulating miRNA model as determined by Pearson correlation coefficient analysis. (F) Increased IGF1 and JUN positivity were more frequently observed in tumor samples of patients in the high‐risk group (*n* = 26) than in the low‐risk group (*n* = 26). Statistical significance was assessed by chi‐square test. (G) Bioinformatics analysis predicted potential binding sites of miR130b on the promotor region of IGF1. (H) The effect of miR130b on transcriptional activity of the IGF1 promoter revealed by luciferase reporter assay in HEK‐293T cells transfected with control mimics or miR130b mimics. Mean ± SD from triplicates is shown by vertical bars (*n* = 3). Statistical significance was assessed by*t*‐test. (I) GSEA analysis of signaling pathways significantly altered in the high and low Ras groups.

MicroRNA can regulate gene expression by targeting mRNA at the 3′‐untranslated region. To determine the role of the 4‐circulating miRNA model in regulation of genes involved in oncogenic signaling pathways, we next performed RNA‐sequencing on 52 patients in the training cohort with available frozen tumor samples and among the bottom 25% of the low‐risk and the top 25% of the high‐risk group of the 4‐circulating miRNA model. Using mirpath v.3 software (Oxford University, Oxford, UK), 214 signaling pathways were identified associated with miR21, miR130b, miR155 and miR28. Using gene ontology analysis of RNA‐sequencing data, 190 signaling pathways were differentially expressed between the low‐risk and the high‐risk groups of the 4‐circulating miRNA model (Fig. 4A). Eleven signaling pathways were found in both mirpath v.3 and gene ontology analysis. The significant lymphoma‐associated pathways were Ras protein signal transduction, cytokine‐mediated signaling pathway, positive regulation of apoptotic process, angiogenesis, negative regulation of cell proliferation, and innate immune response (Fig. 4B). Gene ontology showed that 67 genes were involved in Ras protein signal transduction. Among them, 23 genes were targeted by at least one of the four miRNA and further selected for gene–gene interaction analysis (Fig. 4C). As shown in Fig. 4C, IGF1 and JUN played central roles in Ras protein signal transduction. The cytoscape (Cytoscape Consortium, Seattle, WA, USA) program was used to visualize the interaction of 23 genes involved in Ras protein signal transduction; greater nodes indicated genes that were more likely to be functionally related. IGF1 and JUN N‐terminal kinases (JNK) are major conserved pathways involved in Ras protein signal transduction and overexpressed in DLBCL. Meanwhile, network analysis (Fig. 4D) showed that IGF1 was significantly related to RRAS, FGF2, PLD1, TP53, DOK1, CNKSR1, DHCR24, RIT1, NTN1, PARK7, SHC1, RALGDS, CDKN1A, RB1, SOS1, NF1 and CDKN2A expression, and JUN was related to SHC1, RALGDS, CDKN1A, RB1, SOS1, NF1, CDKN2A, GRAP2, MRAS, ADRA2A, LAT, RRAS, FGF2, PLD1 and TP53 expression. Further analysis demonstrated that IGF1 and JUN displayed significant correlation with the 4‐circulating miRNA model and each miRNA (IGF1, *R* = 0.408, *P* = 0.006, *R* = 0.427, *P* = 0.002, *R* = 0.225, *P* = 0.079, *R* = −0.410, *P* = 0.003, and *R* = 0.371, *P* = 0.007; JUN, *R* = 0.331, *P* = 0.019, *R* = 0.297, *P* = 0.034, *R* = 0.351, *P* = 0.018, *R* = −0.152, *P* = 0.158 and *R* = 0.445, *P* < 0.001; Fig. 4E). Immunohistochemistry was performed to show the protein levels of IGF1 and JUN in tumor samples of 52 DLBCL patients. IGF1 and JUN were more frequently observed in the high‐risk group than in the low‐risk group (*P* = 0.016 and *P* = 0.020, Fig. 4F). Therefore, the 4‐circulating miRNA prognostic model was significantly associated with IGF1 and JUN expression in DLBCL. IGF1 was an established target for miR21 and miR28 [19, 20], and JUN for miR21 and miR155 [21, 22]. Since there is no previous report on miR130b, bioinformatics analysis predicted potential binding sites of IGF1 promoter with miR130b (Fig. 4G). Luciferase reporter assay was performed and showed that miR130b positively regulated the transcriptional activity of the IGF1 promoter region (−674 to −667 bp) in HEK‐293T cells (*P* = 0.001, Fig. 4H). GSEA analysis (Fig. 4I) revealed that the immune‐related pathway was significantly downregulated in the Ras high‐risk group, as compared with the Ras low‐risk group, including immune system development (*P* = 0.015), innate immune response (*P* = 0.030), regulation of cytokine production involved in immune response (*P* = 0.008) and regulation of humoral immune response (*P* = 0.028). Therefore, IGF1 and JUN play important roles in dysregulation of Ras protein signal transduction, which may be related to aberrant tumor immunity in DLBCL.

### 3.6 Association of the 4‐circulating miRNA prognostic model with Ras‐mediated tumor immunity

**Fig. 5:**
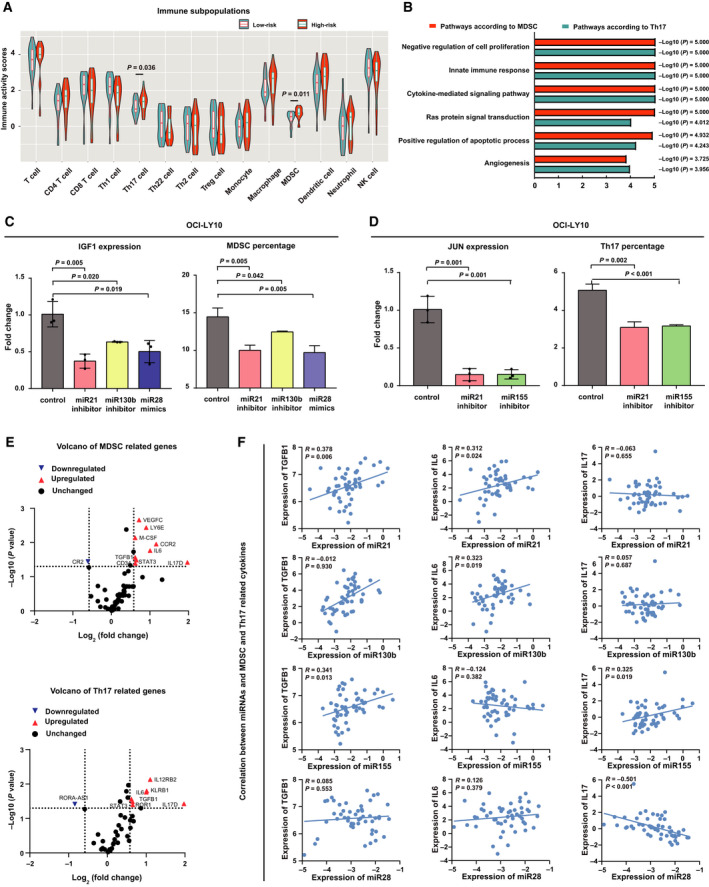
Association of the 4‐circulating miRNA prognostic model with tumor immunity. (A) Immune activity scores of immune cell subsets according to low‐risk and high‐risk group of the 4‐circulating miRNA model. (B) Signaling pathways related to both MDSC and Th17 cells according to gene ontology. (C) Correlation of miRNA with IGF1 expression and MDSC percentage in co‐culture of OCI‐LY10 cells with PBMC. Mean ± SD from triplicates is shown by vertical bars (*n* = 3). Statistical significance was assessed by*t*‐test. (D) Correlation of miRNA with JUN expression and Th17 cells percentage in co‐culture of OCI‐LY10 cells with PBMC. Mean ± SD from triplicates is shown by vertical bars (*n* = 3). Statistical significance was assessed by*t*‐test. (E) Volcano plot of gene expression profile involved in MDSC and Th17 cells, including cytokines and growth factors, intracellular signaling factors, phenotyping expression in MDSC (upper panel) and Th17 cells (lower panel). (F) Correlation of miRNA with TGFB1, IL‐6 and IL‐17D expression.

Immune activity scores of immune cells were analyzed by TIP analysis, including T‐cell subsets, monocytes, macrophages, MDSC, dendritic cells, neutrophil and natural killer cells. MDSC and Th17 cells were significantly higher in the high‐risk group than in the low‐risk group of the 4‐circulating miRNA model (*P* = 0.011 and *P* = 0.036, Fig. 5A). Immune activity score was used to differentiate the low MDSC and the high MDSC groups, as well as the low Th17 and the high Th17 groups. The median immune activity score of MDSC was 0.595 in DLBCL. The patients with immune activity scores over or equal to the median value were regarded as the high MDSC group and those below the median value as the low MDSC group. The median immune activity score of Th17 was 1.266 in DLBCL. The patients with Th17 immune activity score over or equal to the median value were regarded as the high Th17 group and those below the median value as the low Th17 group. Using gene ontology analysis of RNA‐sequencing data, differentially expressed signaling pathways were displayed between the low MDSC and the high MDSC groups, as well as between the low Th17 and the high Th17 groups. Significant lymphoma‐associated pathways included negative regulation of cell proliferation, innate immune response, cytokine‐mediated signaling pathway, Ras protein signal transduction, positive regulation of apoptotic process, and angiogenesis (Fig. 5B), indicating the association of the 4‐circulating miRNA prognostic model with Ras‐mediated tumor immunity. To further confirm the biological effect of 4‐circulating miRNA on tumor immunity, B‐lymphoma cells were transfected with inhibitors of miR21, miR130b and miR155, and mimics of miR28. Mimicking the situation of the patients, lymphoma cells were co‐cultured with PBMC. In the co‐culture system, compared with control cells, knockdown of miR21 and miR130b and overexpression of miR28 significantly decreased IGF1 expression (*P* = 0.005, *P* = 0.020 and *P* = 0.019, respectively) and MDSC percentage (*P* = 0.005, *P* = 0.042 and *P* = 0.005, Fig. 5C). Compared with control cells, knockdown of miR21 and miR155 decreased JUN expression (*P* = 0.001 and *P* = 0.001) and the percentage of Th17 cells (*P* = 0.002 and *P* < 0.001, Fig. 5D). To investigate the biological function of 4‐circulating miRNA on IGF1 and JUN, B‐lymphoma cells were simultaneously transfected with inhibitors of miR21, miR130b and miR155, and mimics of miR28. Western blot showed that knockdown of miR21, miR130b and miR155, and overexpression of miR28 significantly decreased IGF1 and JUN expression, as compared with the control cells (Fig. S2A). Immunofluorescence assay further confirmed that expression levels of IGF1 and JUN were downregulated when B‐lymphoma cells were simultaneously transfected with inhibitors of miR21, miR130b and miR155, and mimics of miR28 (Fig. S2B). MDSC‐ and Th17‐associated genes were differently expressed in high‐risk and low‐risk patients (Fig. 5E). Among MDSC‐associated genes, cytokines and growth factors (M‐CSF, TGFB1, VEGFC, IL‐6 and IL‐17D), phenotyping genes (CCR2, LY6E, CD34 and CR2), and intracellular signaling factors (STAT3) were significantly altered. Similarly, Th17‐associated genes were also involved, such as cytokines and growth factors (TGFB1, IL‐6 and IL‐17D), phenotyping genes (IL12RB2 and KLRB1) and intracellular signaling factors (STAT3, ROR and RORA‐AS1). In DLBCL patients, there was a significant correlation between serum miR21 and TGFB1 (*R* = 0.378, *P* = 0.006) and IL‐6 expression (*R* = 0.312, *P* = 0.024), between serum miR130b and IL‐6 expression (*R* = 0.323, *P* = 0.019), between serum miR155 and TGFB1 expression (*R* = 0.341, *P* = 0.013) and IL‐17D expression (*R* = 0.325, *P* = 0.019), and between serum miR28 and IL‐17D expression (*R* = −0.501, *P* < 0.001, Fig. 5F). These findings showed that the 4‐circulating miRNA model was linked to the interaction of B‐lymphoma cells with immunosuppressive components of tumor microenvironment in DLBCL.

## 4 Discussion

MicroRNA are critically involved in lymphoma progression. This is, to our knowledge, the first miRNA prognostic model to predict effectively relapse and survival in DLBCL. Among the four miRNA predictors, miR21, miR155, and miR28 have been previously included in a distinct miRNA prognostic signature in DLBCL [7, 23, 24]. MicroR130b, though not reported in DLBCL, has been identified as an oncogenic miRNA in adult T‐cell leukemia/lymphoma [25]. Moreover, the 4‐circulating miRNA prognostic model was based on serum miRNA levels, and stable on cohorts varied from clinical features, thus proving to be an easy‐to‐use and efficient prognostic model of DLBCL.

MicroRNA are transcriptionally regulated by TF in hematopoietic cells [26] or, alternatively, exert biological function through modulating genes involved in oncogenic signaling pathways. Here we demonstrated that the 4‐circulating miRNA prognostic model was independent on mutational status of TF, suggesting their regulatory role on oncogenic signaling pathways. Indeed, the high‐risk group of the 4‐circulating miRNA prognostic model was characterized by activation of Ras protein signal transduction. IGF1 and JUN were two key regulators of Ras cascade, and enhanced lymphoma development [27, 28, 29]. As mechanism of action, IGF1 is positively regulated by miR21 and negatively regulated by miR28 [19, 30]. JUN is positively regulated by miR21 and miR155 [21, 31]. We provided *in vitro* and *in vivo* evidence that the four miRNA of the prognostic model modulated Ras protein signal transduction via IGF1 and JUN, indicating an alternative mechanism for oncogenic signaling and tumor progression in DLBCL.

MDSC and Th17 cells are two major subtypes of immunosuppressive cells associated with tumor immunity and are related to poor clinical outcome in B‐cell lymphoma [32, 33]. In solid tumors, miR21 enhances MDSC expansion via TGF‐β and IL‐6 activation [34], and miR130b induces immunosuppressive function of MDSC [35]. MicroR21 promotes Th17 cell accumulation by activating TGF‐β signaling [36], and miR155 induces Th17 cell differentiation by increasing IL‐17 secretion [37]. Moreover, cytokines and growth factors such as TGFB1, IL‐6 and IL‐17D can increase the interplay between MDSC and Th17 cells in tumor microenvironment [38]. Here we showed that the 4‐circulating miRNA of the prognostic model were associated with induction of MDSC and Th17 cells, as well as cytokines TGFB1, IL‐6 and IL‐17D involved in MDSC‐Th17 interaction. Therefore, the 4‐circulating miRNA prognostic model may lead to immune suppression in DLBCL through modulating MDSC and Th17 cells. This information will help in developing potential therapeutic strategies by harnessing tumor microenvironment, thus targeting tumor progression and improving the clinical outcome of DLBCL patients.

## 5 Conclusions

Our findings confirmed that the 4‐circulating miRNA prognostic model enables accurate prediction of disease relapse and prognosis of DLBCL patients and a better understanding of the role of tumor microenvironment in lymphoma progression, making this a potentially valuable biomarker signature in clinical practice for risk stratification in DLBCL.

## Conflict of interest

The authors declare no conflicts of interest.

## Author contributions

RS performed the experiments. ZZ, LW and SC analyzed clinical data. QS, BQ, DF, CL, YZ, JY and AJ provided technical support. W‐LZ designed the study, and directed and supervised the research. ZZ and W‐LZ wrote the manuscript.

## Supporting information


**Fig. S1.** Association of 4‐circulating miRNA prognostic model with genomic alterations.

**Fig. S2.** Association of IGF1 and JUN expression with 4‐miRNA.

**Table S1.** Clinical characteristics in the discovery cohort of patients with DLBCL.

Supplementary Material

## Data Availability

The materials used and datasets used or analyzed in this study are available from the corresponding author on reasonable request.
