# Supplementary figures and images for: A novel prognostic model based on four circulating miRNA in diffuse large B‐cell lymphoma: implications for the roles of MDSC and Th17 cells in lymphoma progression
Source: Mol Oncol. 2020 Nov 9;15(1):246–61. doi: 10.1002/1878-0261.12834 (PMC7782091; doi:10.1002/1878-0261.12834)

A

Gene mutation pattern (n=223)

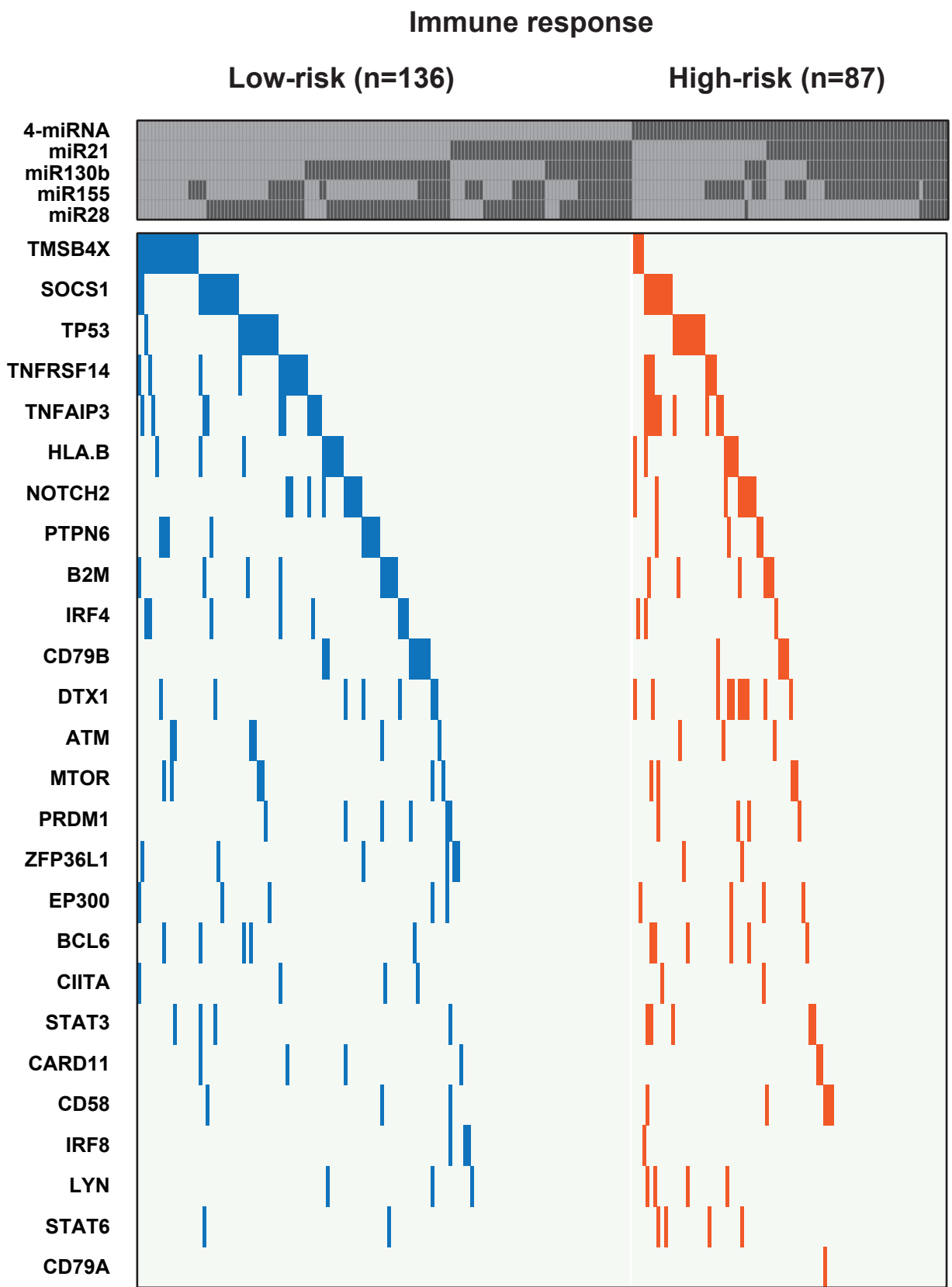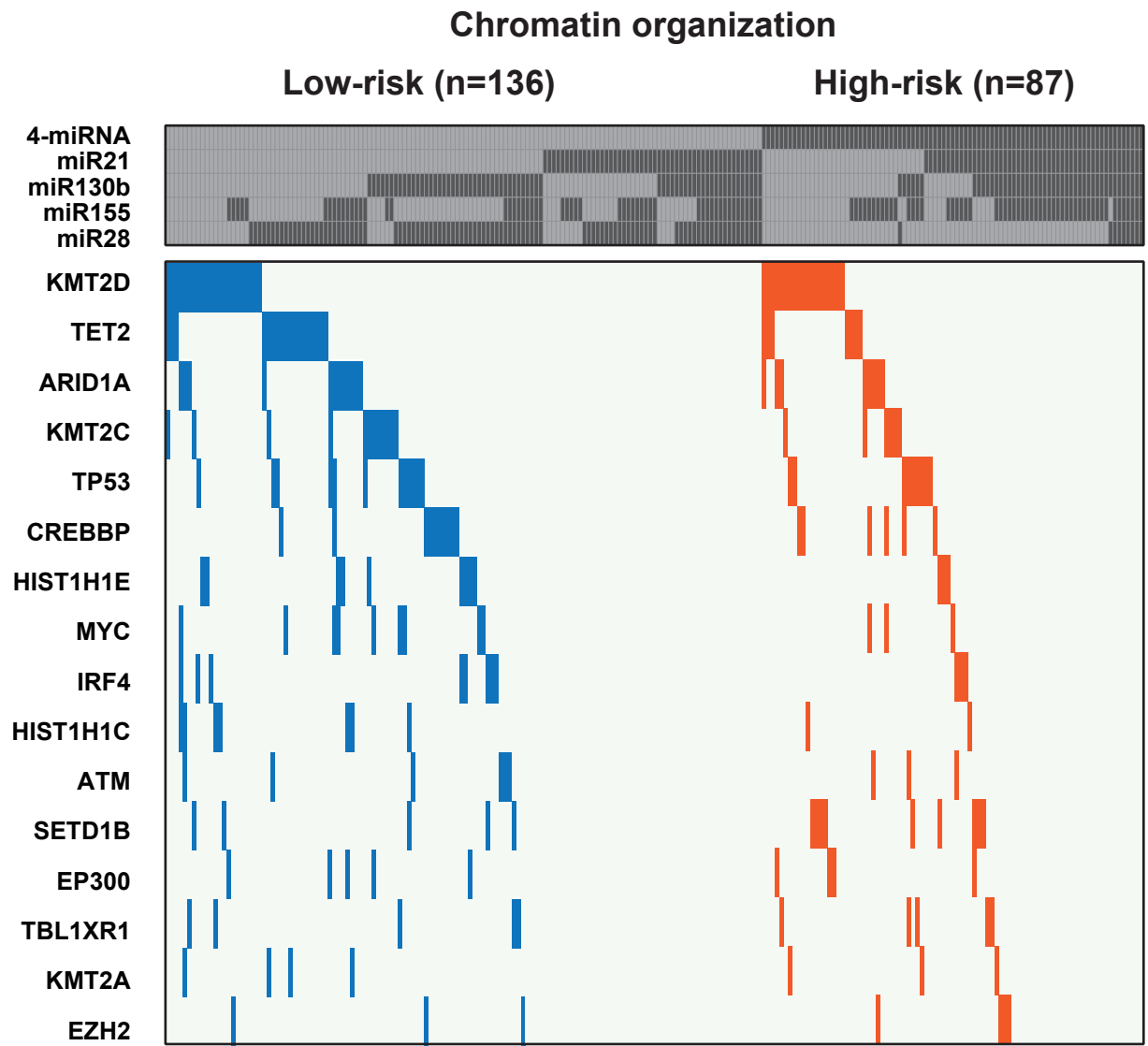

Signaling pathway

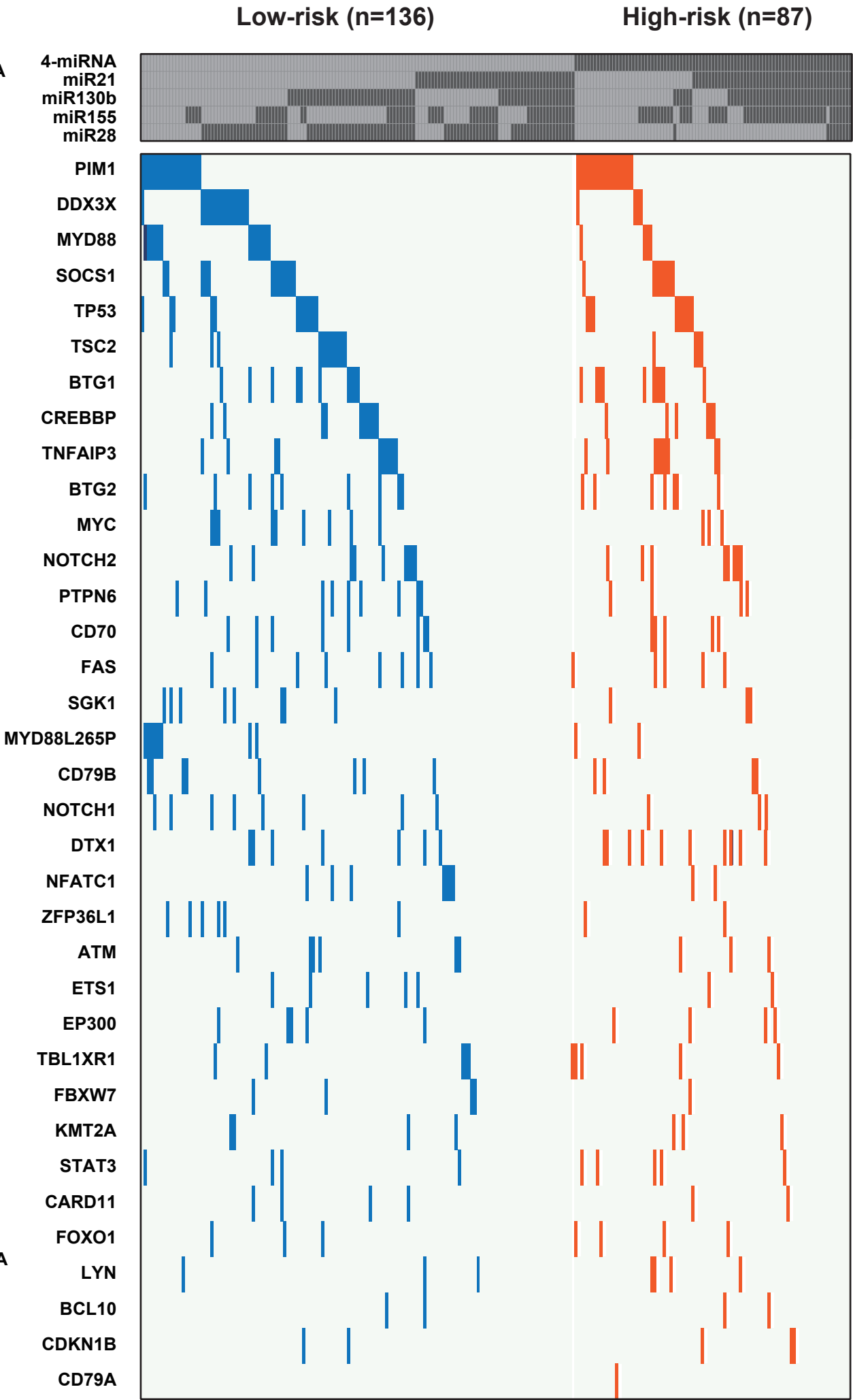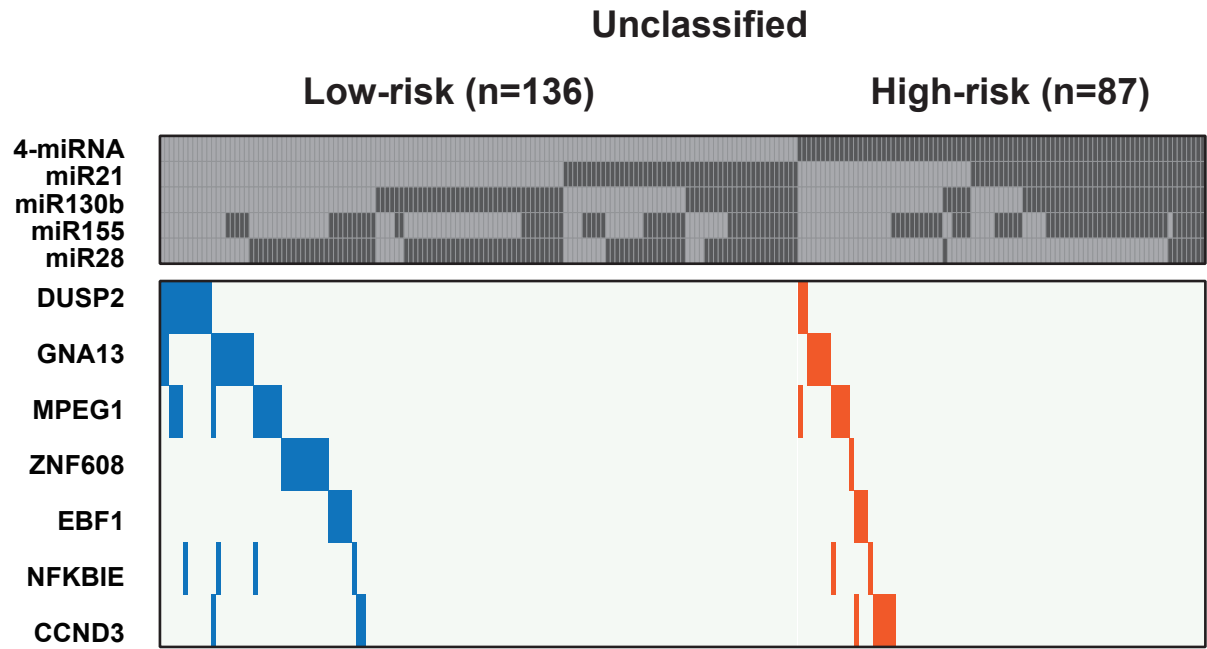

B

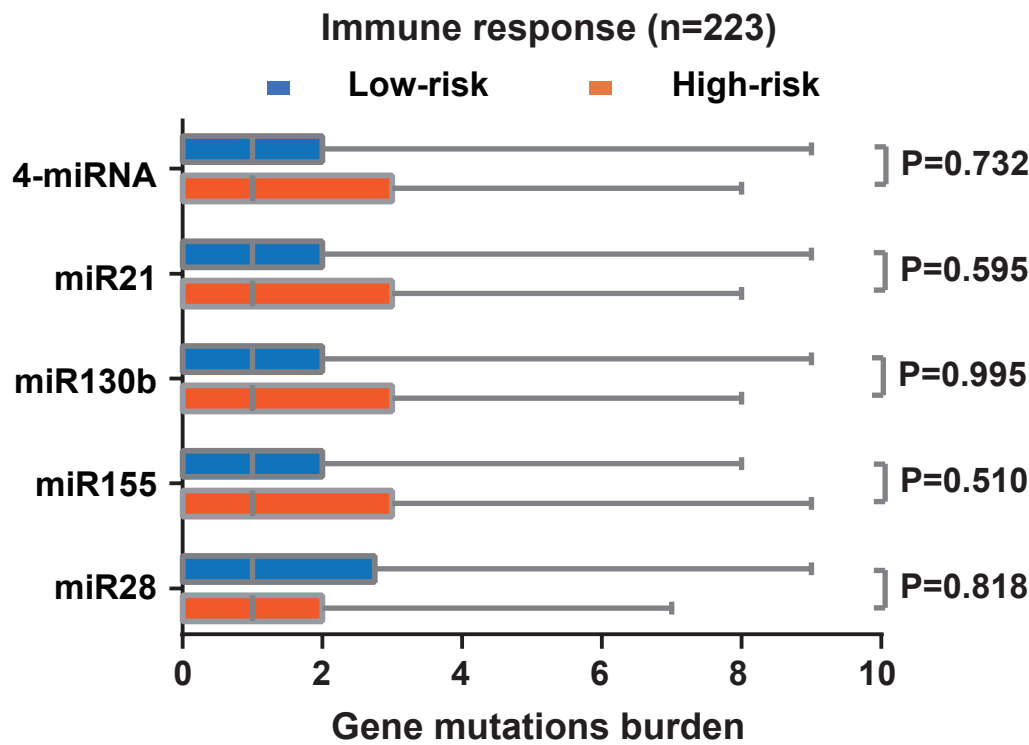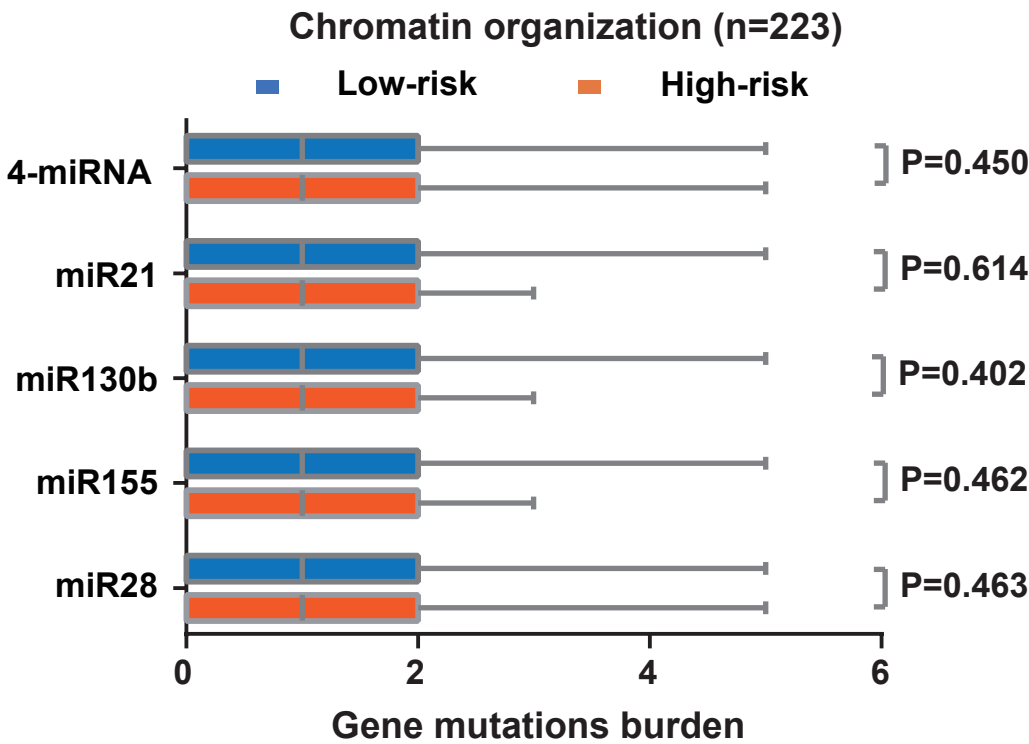

C

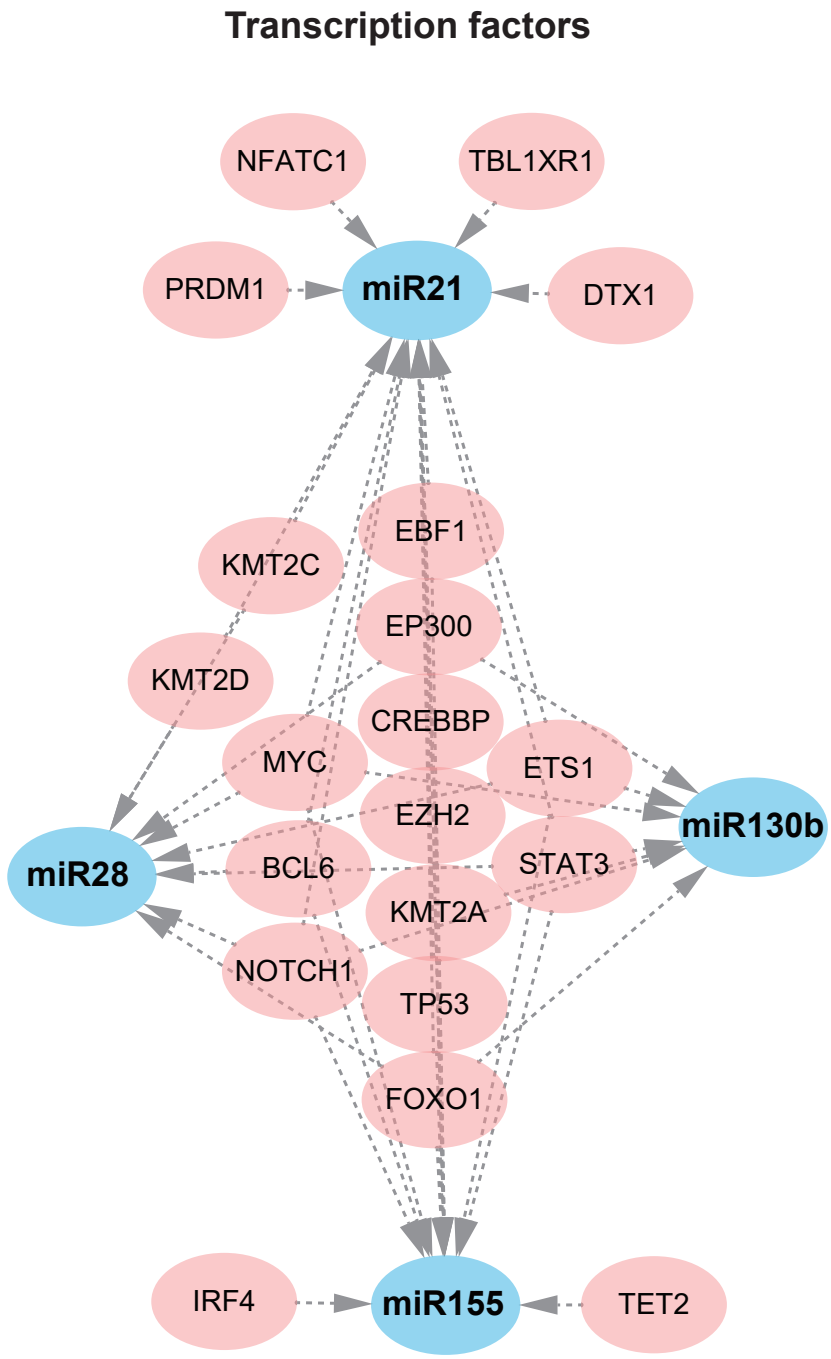

D

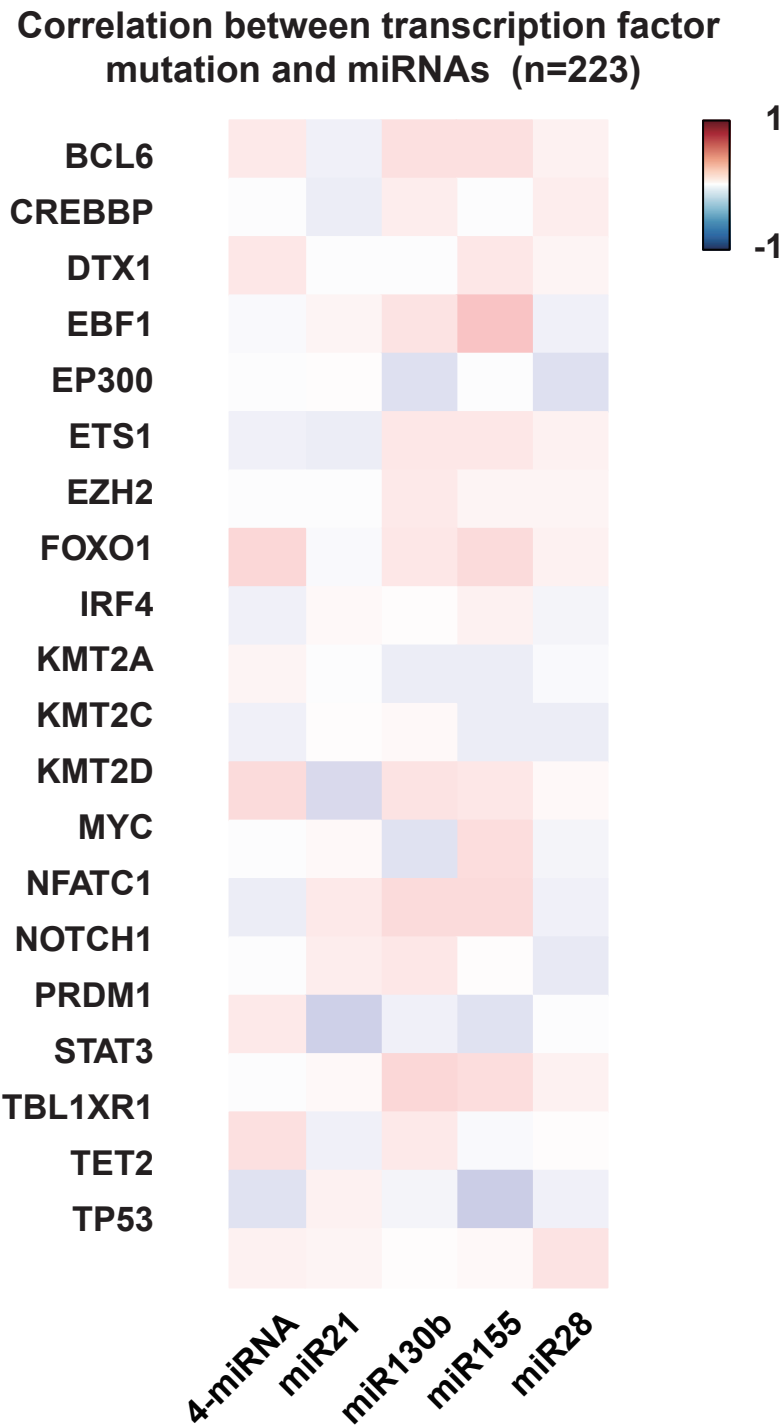

Supplement: Supplementary file 1 — Fig. S1. Association of 4‐circulating miRNA prognostic model with genomic alterations. [file MOL2-15-246-s001.pdf]

## Sun et al. Supplementary Figure S2

**A**

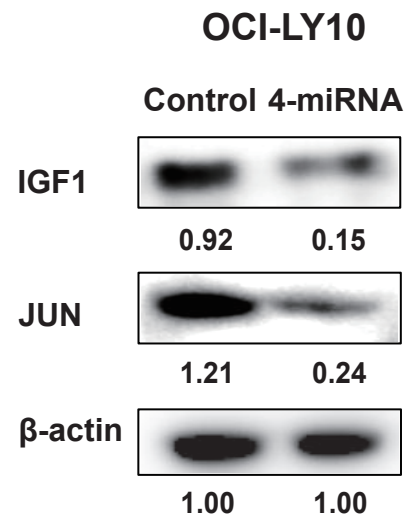

**B**

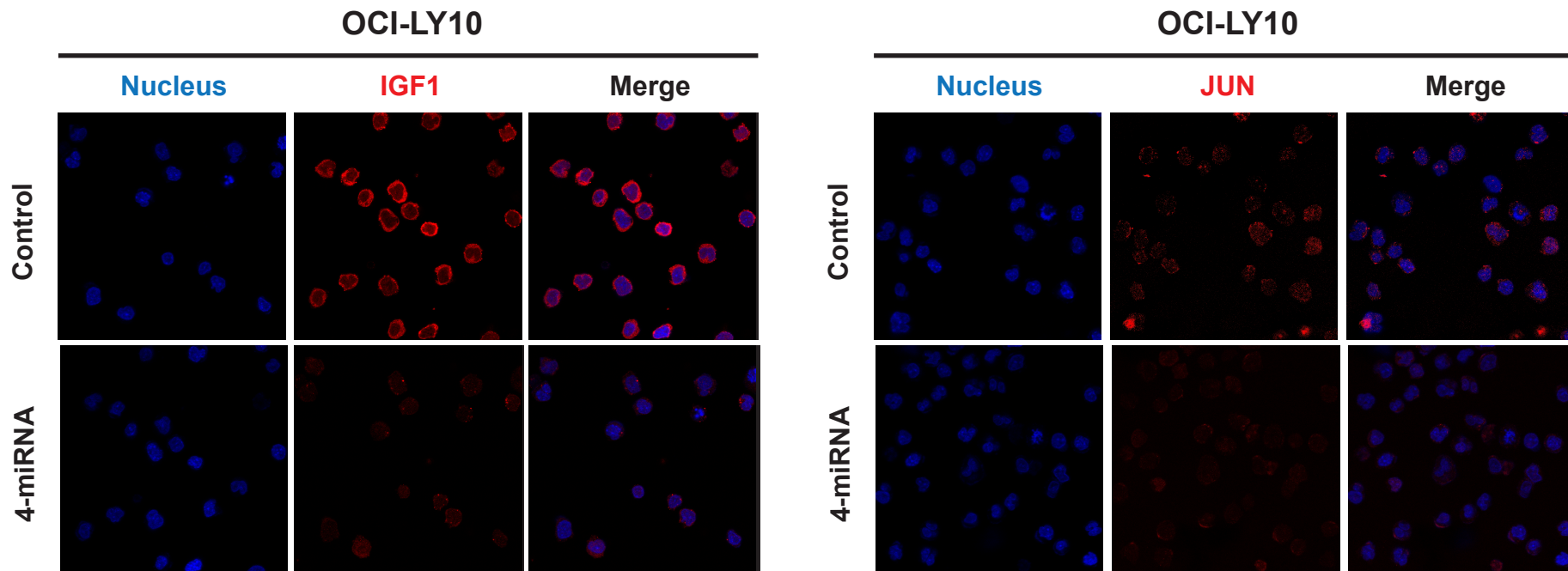

Supplement: Supplementary file 2 — Fig. S2. Association of IGF1 and JUN expression with 4‐miRNA. [file MOL2-15-246-s002.pdf]
