# Supplementary material for: A novel prognostic model based on four circulating miRNA in diffuse large B‐cell lymphoma: implications for the roles of MDSC and Th17 cells in lymphoma progression
Source: Mol Oncol. 2020 Nov 9;15(1):246–61. doi: 10.1002/1878-0261.12834 (PMC7782091; doi:10.1002/1878-0261.12834)
Supplement: Supplementary file 3 — Table S1. Clinical characteristics in the discovery cohort of patients with DLBCL. [file MOL2-15-246-s003.docx]

**Supplementary Table 1. Clinical characteristics in the discovery cohort of patients with DLBCL.**

| Characteristics | Discovery cohort (n=20) |
| --- | --- |
|  |  |
| Sex | |
| Female | 11/20 (55.0%) |
| Male | 9/20 (45.0%) |
| Age | |
| > 60 years | 9/20 (45.0%) |
| ≤ 60 years | 11/20 (55.0%) |
| ECOG | |
| 0-1 | 15/20 (75.0%) |
| 2 | 5/20 (25.0%) |
| Ann Arbor | |
| I-II | 7/20 (35.0%) |
| III-IV | 13/20 (65.0%) |
| Extranodal involvement | |
| No | 12/20 (60.0%) |
| Yes | 8/20 (40.0%) |
| LDH | |
| Normal | 8/20 (40.0%) |
| Elevated | 12/20 (60.0%) |
| International Prognostic Index (IPI) | |
| 0-2 | 11/20 (55.0%) |
| 3-5 | 9/20 (45.0%) |
