# Supplementary material for: A novel prognostic model based on four circulating miRNA in diffuse large B‐cell lymphoma: implications for the roles of MDSC and Th17 cells in lymphoma progression
Source: Mol Oncol. 2020 Nov 9;15(1):246–61. doi: 10.1002/1878-0261.12834 (PMC7782091; doi:10.1002/1878-0261.12834)
Supplement: Supplementary file 4 — Supplementary Material [file MOL2-15-246-s004.docx]

**SUPPLEMENTARY INFORMATION TO:**

**A novel 4-circulating miRNA prognostic model in diffuse large B-cell lymphoma: Role of MDSCs and Th17 cells in lymphoma progression.**

Rui Sun^1*^, Zhong Zheng^1*^, Li Wang^1,2^, Shu Cheng^1^, Qing Shi^1^, Bin Qu^3^, Di Fu^1^, Christophe Leboeuf^4^, Yan. Zhao^1^, Jing Ye^2^, Anne Janin^4^, Wei-Li Zhao^1,2, †^

^*^ These authors contributed equally to this work.

^1^Shanghai Institute of Hematology, State Key Laboratory of Medical Genomics, National Research Center for Translational Medicine at Shanghai, Ruijin Hospital Affiliated to Shanghai Jiao Tong University School of Medicine, Shanghai, China

^2^Pôle de Recherches Sino-Français en Science du Vivant et Génomique, Laboratory of Molecular Pathology, Shanghai, China

^3^Department of Laboratory Medicine, Shanghai Rui Jin Hospital, Shanghai Jiao Tong University School of Medicine, Shanghai, China

^4^U1165 Inserm/Université Paris 7, Hôpital Saint Louis, Paris, France

^†^ Corresponding author: Prof. Wei-Li Zhao, Shanghai Institute of Hematology, State Key Laboratory of Medical Genomics, National Research Center for Translational Medicine at Shanghai, Ruijin Hospital Affiliated to Shanghai Jiao Tong University School of Medicine, 197 Rui Jin Er Road, Shanghai 200025, China. Tel: 0086-21-64370045, Fax: 0086-21-64743206. Email: zhao.weili@yahoo.com.

**Supplementary Table 1. Clinical characteristics in the discovery cohort of patients with DLBCL.**

| Characteristics | Discovery cohort (n=20) |
| --- | --- |
|  |  |
| Sex | |
| Female | 11/20 (55.0%) |
| Male | 9/20 (45.0%) |
| Age | |
| > 60 years | 9/20 (45.0%) |
| ≤ 60 years | 11/20 (55.0%) |
| ECOG | |
| 0-1 | 15/20 (75.0%) |
| 2 | 5/20 (25.0%) |
| Ann Arbor | |
| I-II | 7/20 (35.0%) |
| III-IV | 13/20 (65.0%) |
| Extranodal involvement | |
| No | 12/20 (60.0%) |
| Yes | 8/20 (40.0%) |
| LDH | |
| Normal | 8/20 (40.0%) |
| Elevated | 12/20 (60.0%) |
| International Prognostic Index (IPI) | |
| 0-2 | 11/20 (55.0%) |
| 3-5 | 9/20 (45.0%) |

**Supplementary Figure S1. Association of 4-circulating miRNA prognostic model with genomic alterations.**

**A:** Gene mutations identified by WES, WGS, and targeted sequencing in DLBCL (n=223). **B:** Relation of tumor mutation burden with each miRNA and 4-miRNA prognostic model. **C:** Schematic graph of transcription factors with each miRNA. **D:** Relation of transcription factor mutation with each miRNA and 4-miRNA prognostic model.

**Supplementary Figure S2. Association of IGF1 and JUN expression with 4-miRNA.**

**A:** IGF1 and JUN expression detected by western blot in B-lymphoma cells simultaneously transfected with inhibitor of miR21, miR130b, miR155, and mimics of miR28. **B:** IGF1 and JUN expression detected by immunofluorence in B-lymphoma cells simultaneously transfected with inhibitor of miR21, miR130b, miR155, and mimics of miR28 (Representative immunofluorescene images of IGF1[red], JUN[red] with nucleus counterstained with DAPI [blue]).
